# The Association Between Hyperuricemia and Obesity Metabolic Phenotypes in Chinese General Population: A Retrospective Analysis

**DOI:** 10.3389/fnut.2022.773220

**Published:** 2022-04-18

**Authors:** Xiaojing Feng, Yanyi Yang, Huiqi Xie, Siqi Zhuang, Yiyuan Fang, Yufeng Dai, Ping Jiang, Hongzhi Chen, Haoneng Tang, Lingli Tang

**Affiliations:** ^1^Department of Laboratory Medicine, The Second Xiangya Hospital, Central South University, Changsha, China; ^2^Health Management Center of the Second Xiangya Hospital, Central South University, Changsha, China; ^3^National Clinical Research Center for Metabolic Disease, The Second Xiangya Hospital, Central South University, Changsha, China; ^4^Key Laboratory of Diabetes Immunology, Ministry of Education, Metabolic Syndrome Research Center, Department of Metabolism and Endocrinology, The Second Xiangya Hospital, Central South University, Changsha, China

**Keywords:** obesity, uric acid, hyperuricemia, metabolic phenotypes, overweight

## Abstract

**Purpose:**

Serum uric acid (UA) not only affects the development of obesity but also alters the metabolic status in obese subjects; thus we investigated the relationship between serum UA and the overweight/obese metabolic phenotypes.

**Methods:**

The demographic, biochemical, and hematological data were collected for 12,876 patients undergoing routine physical examination, and 6,912 participants were enrolled in our study. Participants were classified into four obesity metabolic phenotypes according to their BMI and the presence of metabolic syndrome: metabolically healthy overweight/obese (MHOO), metabolically healthy and normal weighted (MHNW), metabolically abnormal and overweight/obese (MAOO), and metabolically abnormal but normal weighted (MANW). Univariate and multivariate logistic regression analysis, stratified analysis, and also interaction analysis were conducted to analyze the relationship between serum UA and obesity metabolic phenotypes.

**Results:**

Multivariable logistic regression analysis showed that hyperuricemia was positively associated with MHOO, MANW, and MAOO phenotypes relative to MHNW. After adjusting for the confounding factors, the odds ratios (OR) for individuals with hyperuricemia to be MHOO, MANW, and MAOO phenotypes were 1.86 (1.42–2.45), 2.30 (1.44–3.66), and 3.15 (2.34–4.24), respectively. The ORs for having MHOO, MANW, and MAOO increased 6% [OR: 1.06 (1.05–1.07), *P* < 0.0001], 5% [OR: 1.05 (1.03–1.07), *P* < 0.0001], and 11% [OR: 1.11 (1.10–1.13), *P* < 0.0001] for each 10 unit (μmol/L) of increase in serum UA level. Stratification analysis as well as an interaction test showed that sex and age did not interfere with the association of hyperuricemia with each metabolic phenotype. In terms of the components of the metabolic syndrome, after adjusting for other confounding factors including all of the metabolic indicators except itself, hyperuricemia was positively associated with increased BMI [OR: 1.66 (1.32–2.09), *P* < 0.0001], hypertriglyceridemia [OR: 1.56 (1.21–2.02), *P* = 0.0006], and hypertension [OR: 1.22 (1.03–1.46), *P* = 0.0233], while it had no significant association with hyperglycemia and low HDL-C (all *P* > 0.05).

**Conclusion:**

In our study, we discovered that hyperuricemia was positively associated with MHOO, MANW, and MAOO phenotypes, and this relationship was independent of sex and age.

## Introduction

Obesity has been notorious for being the risk factors of multiple diseases, for instance, metabolic abnormalities (1), cardiovascular disease (2), hypertension (3), lung disease (4), and even female infertility (5). A tremendous increase in health expenses related to obesity has also been reported (6, 7). Therefore, understanding the association between obesity and other diseases is crucial for managing the global pandemic of obesity.

However, within overweight/obese subjects, there are differential metabolic profiles, where many are “fat but fit” while others are typically accompanied with metabolic disorders (8). Additionally, there are people who are “unhealthy but normal weight,” that is, although being normal-weighted, they still developed metabolic disorders (9). Therefore, based on the body mass index (BMI) and the presence of components of metabolic syndrome, the overweight/obese metabolic phenotypes may include metabolically healthy overweight/obese (MHOO), metabolically abnormal overweight/obese (MAOO), and metabolically abnormal but normal weight (MANW), and metabolically healthy and normal weight (MHNW) phenotype (10). Studies have indicated that the metabolic phenotypes have significant differences in inflammation levels (11, 12), and the MHOO phenotype was associated with a lower level of systemic inflammation (13). As for MANW subjects, they are also characterized by higher adipose tissue inflammation level compared to MHNW phenotype (14). These findings suggest a crucial role of inflammation in the development of different obesity metabolic phenotypes, which leads to the question of what factors may alter the inflammation and metabolic status in overweight/obese or normal weight subjects.

Uric acid (UA) is an end product of purine metabolism, and its elevation in serum has been significantly associated with the progression of metabolic syndrome (15). It has been well documented as a pro-inflammatory and pro-oxidant substance (16). These properties of UA may result in endothelial dysfunction that may represent a pathogenic mechanism for coronary disease, diabetes, and hypertension (15). Some studies have found that increased fat accumulation in the liver could be induced by UA through endoplasmic reticulum stress and upregulation of lipogenesis, and these metabolic alterations may result in obesity and diabetes (17–19). Interestingly, in addition to the liver, mature adipocytes and adipose tissues were found to be able to produce UA, which may represent an underlying mechanism of the low-grade inflammation and insulin resistance observed in subjects with metabolic syndrome (20, 21). Research also has shown that serum levels of UA are a significant predictor of unhealthy obesity in youth and adults (22). However, whether this association was consistent in other obesity phenotypes remains unknown. In addition, considering sex differences in UA levels, whether the association was influenced by factors such as sex or age has not yet been fully understood.

Therefore, we hypothesized that hyperuricemia is involved in the progression of obesity and may have different associations with various obesity phenotypes. In this study, we performed a retrospective analysis of the relationship between hyperuricemia and various overweight/obese metabolic phenotypes in the Chinese general population and explored the potential role of UA in the differences of various metabolic states in overweight/obese subjects from an epidemiological interpretation.

## Materials and Methods

### Study Population

This study was approved by the Ethics Committee of Second Xiangya Hospital of Central South University and adhered to the principles of the Declaration of Helsinki. The subjects were participants for physical examination at the Health Management Center of Second Xiangya Hospital of Central South University from January 2019 to December 2019. The total number of the subjects was 12,876, whose age range was from 18 to 75 years. After excluding subjects with missing or erroneous information on covariates, thin (BMI < 18.5 kg/m^2^), abnormal renal function, severe infections, pregnancy, cancers, and those who were not Chinese, a total of 6,912 eligible subjects were included in the final analysis (Supplementary Figure 1).

### Laboratory Measurements

Anthropometric data were collected during the visit for physical examination of the participants. Weight and height were measured according to the recommendations of the World Health Organization, with an accuracy to the nearest 0.1 kg and 0.1 cm respectively, with the participants in light weight clothing without shoes. BMI was calculated as weight/height^2^ (kg/m^2^). Blood pressure was measured twice using a digital sphygmomanometer according to the standard protocol in a sitting resting position after at least 5 min of rest.

Blood samples from participants who underwent overnight fasting were collected in the morning and analyzed within an hour in the hospital. The hepatic parameters including alanine aminotransferase (ALT), aspartate aminotransferase (AST), direct bilirubin (DBIL), albumin, total protein (TP), the renal parameters including urea, creatinine (Cr), the lipid parameters including total cholesterol (TC), triglyceride (TG), high-density lipoprotein cholesterol (HDL-C), low-density lipoprotein cholesterol (LDL-C), and UA were measured by Abbott C16000 analyzers (Abbott, Chicago, IL, United States). Fasting plasma glucose (FPG) was measured using the glucose oxidase-peroxidase method in Abbott C16000 analyzers (Abbott, Chicago, IL, United States). Glycated hemoglobin (HbA1c) was measured with high-performance liquid chromatography using Arkray HA-8160 analyzers (Arkray, Tokyo, Japan). The hematological parameters including white cell count (WBC), neutrophil (NEUT), eosinophils (EO), red cell count (RBC), hemoglobin (HGB), hematocrit (HCT), mean corpuscular volume (MCV), mean corpuscular hemoglobin (MCH), and mean corpuscular hemoglobin concentration (MCHC) were detected by Sysmex XN counters (Sysmex, Kobe, Japan).

### Definition of Overweight/Obesity, Metabolic Syndrome, Metabolic Phenotypes, and Hyperuricemia

Overweight/obesity is defined as a BMI ≥23 kg/m^2^ (23). Metabolic abnormalities were identified according to criteria established by the Adult Treatment Panel III of the National Cholesterol Education Program (NCEP-ATPIII) (24, 25). Participants with two or more of the following four components were considered metabolically abnormal: (1) systolic blood pressure (SBP) ≥130 mmHg or diastolic blood pressure(DBP) ≥85 mmHg (elevated blood pressure); (2) FPG ≥ 5.60 mmol/L (elevated FPG); (3) TG ≥1.7 mmol/L (elevated TG); and (4) HDL-C <1.04 mmol/L in men or <1.29 mmol/L in women (low HDL-C) (24, 25). Overweight/obese metabolic phenotypes were defined according to the presence/absence of overweight/obesity and metabolic abnormalities, and all study subjects were classified into four groups: MHNW, MANW, MHOO, and MAOO. Hyperuricemia was defined as serum UA level >428 μmol/L in men or >357 μmol/L in women.

### Statistical Analysis

Continuous variables with normal distribution were expressed as mean [standard deviation (SD)] and those with non-normal distribution as median (IQR: 25–75%), while categorical variables were reported as numbers and percentages (%). Differences in continuous variables between the groups were tested using *t*-test for normally distributed variables and Mann–Whitney U tests for non-normally distributed variables. Differences in categorical variables were analyzed by Chi-square test. The relationship between UA levels and clinic–metabolic parameters was analyzed by the Spearman’s correlation coefficient as well as partial correlation analysis.

Multivariable logistic regression was used to estimate the association of hyperuricemia or UA serum levels (scaled to 10 μmol/L increments) with overweight/obese metabolic phenotypes and its related metabolic indexes. Confounders were screened according to the *P* value when introducing different indexes into the regression models, and indexes with *P* value less than 0.1 were included as covariates. Non-adjusted and adjusted models were used to assess confounding. In addition, stratified analyses and interaction analyses by sex, age (<45 years, 45–60 years, ≥60 years) were further conducted.

All statistical analyses were performed using SPSS version 20.0 (SPSS Inc., Chicago, IL, United States), software Empower (R)^1^ (X & Y Solutions Inc., Boston, MA, United States), and R.^2^ Two-tailed *P* < 0.05 was considered statistically significant.

## Results

### Baseline Characteristics of the Study Population

A total of 6,912 participants were enrolled in our study. Table 1 shows the baseline characteristics of the participants. The percentages of men and women were close (49.83 and 50.17%), with an average age of 48.88 ± 11.85 years. All participants were classified into two groups according to UA levels: normouricemia group and hyperuricemia group. Significant differences were observed in sex composition between the two groups (*P* < 0.001), where the hyperuricemia group mainly consisted of men (72.14 vs 27.86%), while in the normouricemia group it was similar (47.04 vs 52.96%). The two groups had no significant difference in age but the BMI of the hyperuricemia group was significantly higher than those with normal UA levels (*P* < 0.001). The average or median value of metabolic indicators (i.e., SBP, DBP, FPG, TG, TC, HDL-C, and LDL-C) in the hyperuricemia group was significantly different from the normouricemic group (all *P* < 0.05). All of the other laboratory indicators were also significantly different between the two groups (all *P* < 0.001) except pulse, HbA1c, DBIL, MCV, and MCH.

**TABLE 1:** Baseline characteristics of the study population.

Characteristics	All (*n* = 6,912)	Serum UA level		*P* value*
		Normouricemia (*n* = 6,144)	Hyperuricemia (*n* = 768)	
**Sex**				
Male	3444 (49.83%)	2890 (47.04%)	554 (72.14%)	<0.001
Female	3468 (50.17%)	3254 (52.96%)	214 (27.86%)	
Age, years	48.88 (11.85)	48.97 (11.77)	48.17 (12.39)	0.077
<45	2443 (35.34%)	2159 (35.14%)	284 (36.98%)	0.545
45–60	3332 (48.21%)	2967 (48.29%)	365 (47.53%)	
≥60	1137 (16.45%)	1018 (16.57%)	119 (15.49%)	
BMI, kg/m^2^	24.36 (3.13)	24.13 (3.04)	26.22 (3.28)	<0.001
Pulse, bpm	77.66 (10.99)	77.66 (10.97)	77.68 (11.11)	0.962
SBP, mmHg	124.29 (17.11)	123.59 (17.01)	129.96 (16.88)	<0.001
DBP, mmHg	76.32 (11.30)	75.75 (11.07)	80.86 (12.03)	<0.001
FPG, mmol/L	4.74 (4.39–5.17)	4.73 (4.38–5.16)	4.80 (4.41–5.25)	0.014
TG, mmol/L	1.37 (0.95–2.03)	1.32 (0.92–1.92)	1.96 (1.36–2.93)	<0.001
TC, mmol/L	4.90 (0.95)	4.87 (0.94)	5.14 (0.99)	<0.001
HDL-C, mmol/L	1.31 (0.29)	1.32 (0.29)	1.21 (0.25)	<0.001
LDL-C, mmol/L	2.92 (0.79)	2.90 (0.79)	3.09 (0.78)	<0.001
HbA1c,%	5.60 (5.40–5.90)	5.60 (5.40–5.80)	5.60 (5.40–5.90)	0.972
ALT, U/L	19.00 (13.90–27.90)	18.40 (13.60–26.80)	25.20 (17.90–38.02)	<0.001
AST, U/L	21.40 (18.20–25.70)	21.10 (18.00–25.30)	23.50 (19.80–29.20)	<0.001
TP, g/L	72.56 (3.82)	72.42 (3.79)	73.69 (3.87)	<0.001
ALB, g/L	43.60 (2.38)	43.53 (2.36)	44.19 (2.47)	<0.001
DBIL, U/L	3.30 (2.50–4.20)	3.30 (2.50–4.20)	3.20 (2.60–4.30)	0.860
Cr, μmol/L	67.00 (56.40–79.40)	65.50 (55.60–78.00)	78.65 (67.85–88.30)	<0.001
BUN, mmol/L	4.89 (4.14–5.69)	4.85 (4.10–5.66)	5.12 (4.48–5.83)	<0.001
UA, μmol/L	310.69 (81.15)	292.90 (64.55)	453.05 (56.00)	<0.001
WBC, × 10^9^/L	5.94 (5.04–7.00)	5.88 (4.99–6.93)	6.44 (5.48–7.33)	<0.001
NEUT, × 10^9^/L	3.54 (2.87–4.35)	3.51 (2.84–4.31)	3.76 (3.09–4.61)	<0.001
EO, × 10^9^/L	0.11 (0.07–0.19)	0.11 (0.07–0.18)	0.14 (0.09–0.22)	<0.001
RBC, × 10^12^/L	4.82 (0.53)	4.79 (0.52)	5.05 (0.53)	<0.001
HGB, g/L	143.63 (16.41)	142.66 (16.34)	151.39 (14.79)	<0.001
HCT,%	43.43 (4.26)	43.19 (4.24)	45.32 (3.88)	<0.001
MCV, fL	90.37 (5.84)	90.42 (5.90)	90.00 (5.32)	0.060
MCH, pg	30.20 (29.20–31.20)	30.20 (29.20–31.20)	30.30 (29.30–31.20)	0.194
MCHC, g/L	330.38 (12.14)	329.94 (12.14)	333.85 (11.64)	<0.001

Data are presented as the Mean (SD) or Median (IQR: Q1–Q3) for continuous variables and percentage for categorical variables. *The t-test or Mann–Whitney U test or Chi-square test were used for comparisons between two groups.

### Prevalence of Different Overweight/Obese Metabolic Phenotypes in Hyperuricemia and Normouricemia Groups

**FIGURE 1:**
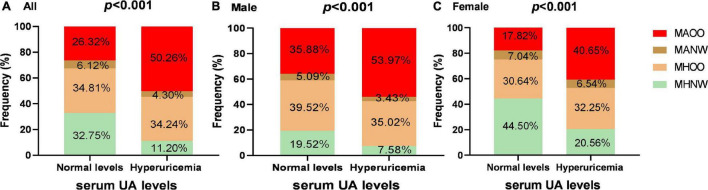
The proportion of obesity metabolic phenotypes among the subjects with hyperuricemia and normal UA levels. Panels **(A–C)** show the proportion differences of various obesity metabolic phenotypes in hyperuricemia group and normouricemic group in all subjects, men and women, respectively. Differences in the proportion of obesity metabolic phenotypes were analyzed by Chi-square test.

The differences in the proportion of obesity metabolic phenotypes were analyzed in the two groups (Figure 1). When compared with the normouricemic group, the percentage of MAOO phenotype was significantly higher in hyperuricemia group (50.26 vs 26.32%, *P* < 0.001), while the percentage of MHNW and MANW phenotypes were significantly lower in the hyperuricemia group (11.20 vs 32.75%, *P* < 0.001; 4.30 vs 6.12%, *P* = 0.004).

We further stratified the participants according to their sex. Among male participants, percentages of MAOO, MHOO, and MHNW phenotypes were significantly different in hyperuricemia group from the normouricemic group (MAOO: 53.97 vs 35.88%, *P* < 0.001; MHOO: 35.02 vs 39.52%, *P* = 0.047; MHNW: 7.58 vs 19.52%, *P* < 0.001), while the percentage was basically consistent in the MANW phenotype. In female participants, the percentage of two phenotypes were different with significance (MAOO: 40.65 vs 17.82%, *P* < 0.001; MHNW: 20.56 vs 44.50%, *P* < 0.001).

### Association of the Presence of Hyperuricemia and the Occurrence of Different Overweight/obese Metabolic Phenotypes

Multivariate logistic regression analyses were conducted to investigate the association between hyperuricemia and metabolic phenotypes (Table 2). The results showed that MHOO, MANW, and MAOO phenotypes were all positively associated with hyperuricemia in all the models when using MHNW phenotype as a reference phenotype. After adjusting for all confounding factors, the OR for individuals with hyperuricemia to be MHOO, MANW, and MAOO phenotypes were 1.86(95%CI:1.42–2.45; *P* < 0.0001), 2.30(95%CI:1.44–3.66; *P* = 0.0005), and 3.15(95%CI:2.34–4.24; *P* < 0.0001), respectively. The association between UA serum levels and each phenotype were also analyzed (Table 2). In the analyses adjusted for all relevant confounders, the OR for having MHOO, MANW, and MAOO phenotypes increased by 6% [OR: 1.06 (1.05–1.07), *P* < 0.0001], 5% [OR: 1.05 (1.03–1.07), *P* < 0.0001] and 11% [OR: 1.11 (1.10–1.13), *P* < 0.0001], respectively, for each 10 unit (μmol/L) of increase in UA levels.

**TABLE 2:** Multivariable logistic regression analysis of the association between hyperuricemia and obesity metabolic phenotypes.

Models	MHNW	MHOO		MANW		MAOO	
		OR (95%CI)	*p* value	OR (95%CI)	*p* value	OR (95%CI)	*p* value
**Hyperuricemia**							
Model 1	1	2.88 (2.24–3.70)	<0.0001	2.05 (1.35–3.11)	0.0007	5.58 (4.38–7.12)	<0.0001
Model 2	1	2.40 (1.85–3.12)	<0.0001	2.21 (1.43–3.42)	0.0004	4.58 (3.53–5.94)	<0.0001
Model 3	1	2.29 (1.76–2.98)	<0.0001	2.37 (1.52–3.68)	0.0001	4.25 (3.25–5.55)	<0.0001
Model 4	1	2.04 (1.56–2.68)	<0.0001	2.43 (1.54–3.84)	0.0001	3.64 (2.74–4.84)	<0.0001
Model 5	1	1.86 (1.42–2.45)	<0.0001	2.30 (1.44–3.66)	0.0005	3.15 (2.34–4.24)	<0.0001
**UA level (Per 10 μmol/L increment)**							
Model 1	1	1.09 (1.08–1.10)	<0.0001	1.05 (1.03–1.06)	<0.0001	1.15 (1.14–1.16)	<0.0001
Model 2	1	1.07 (1.06–1.09)	<0.0001	1.05 (1.03–1.07)	<0.0001	1.13 (1.11–1.14)	<0.0001
Model 3	1	1.07 (1.06–1.08)	<0.0001	1.05 (1.03–1.07)	<0.0001	1.13 (1.11–1.14)	<0.0001
Model 4	1	1.07 (1.05–1.08)	<0.0001	1.06 (1.04–1.08)	<0.0001	1.12 (1.11–1.14)	<0.0001
Model 5	1	1.06 (1.05–1.07)	<0.0001	1.05 (1.03–1.07)	<0.0001	1.11 (1.10–1.13)	<0.0001

*Model 1 was unadjusted; Model 2 was adjusted for sex and age; Model 3 was adjusted for model 2 plus TC, LDL-C, HbA1c; Model 4 was adjusted for model 3 plus ALT, AST, TP, ALB, Urea, Crea; Model 5 was adjusted for model 4 plus WBC, NEUT, EO, RBC, HGB, HCT, MCV, MCH, MCHC.*

**FIGURE 2:**
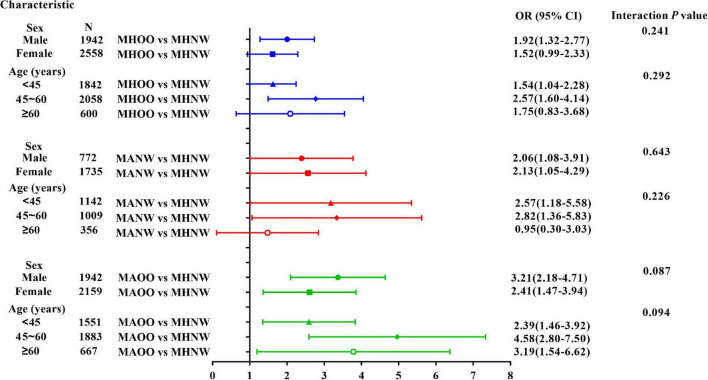
Stratified analyses and interaction tests of the association between hyperuricemia and obesity phenotypes. Models are adjusted for HbA1c, TC, LDL-C, ALT, AST, TP, albumin, Cr, BUN. WBC, NEUT, EO, RBC, HGB, HCT, MCV, MCH, and MCHC.

Stratification analysis and interaction analysis were further performed to explore whether the positive association between hyperuricemia and metabolic phenotypes were influenced by sex or age (Figure 2). Interaction analysis showed that both sex and age did not significantly interfere with the positive association of hyperuricemia and each phenotype (all *P* > 0.05). However, the association between hyperuricemia and these phenotypes were different among different sex and age subgroups. Hyperuricemia was significantly associated with all phenotypes in men (all *P* < 0.05), and was significantly associated with MANW and MAOO phenotypes in women (all *P* < 0.05). As for age stratification, the positive association between the hyperuricemia and MHOO or MANW phenotype was not significant in people older than 60 years (all *P* > 0.05), but were significant in those younger than 60 years (all *P* < 0.05); and hyperuricemia was positively associated with MAOO phenotype in all the age subgroups (all *P* < 0.01).

### Correlation Analysis of UA Serum Levels With Clinical and Laboratory Parameters

We then conducted correlation analysis on UA and clinical and laboratory parameters after uncorrected and corrected for confounders (Table 3), and the Spearman correlation analysis results showed that UA was correlated with all indicators. After adjusting for age and sex, partial correlation analysis also showed that except pulse and MCH, UA was still positively associated with BMI, SBP, DBP, TG, TC, LDL-C, TP, Crea, WBC, RBC, HGB, HCT, while negatively correlated with HDL-C.

**TABLE 3:** Correlations between serum UA levels and anthropometric and laboratory parameters in all individuals.

Variable	UA level^a^		UA level^b^	
	*r*	*P*	*r*	*P*
Age	0.021	0.085	–	–
BMI	0.408	<0.001	0.275	<0.001
Pulse	–0.039	0.001	0.002	0.843
SBP	0.261	<0.001	0.154	<0.001
DBP	0.277	<0.001	0.158	<0.001
TG	0.406	<0.001	0.169	<0.001
TC	0.128	<0.001	0.136	<0.001
HDL-C	–0.334	<0.001	–0.112	<0.001
LDL-C	0.167	<0.001	0.118	<0.001
FPG	0.068	<0.001	–0.050	<0.001
HbA1c	0.118	<0.001	–0.037	0.002
ALT	0.401	<0.001	0.097	<0.001
AST	0.285	<0.001	0.065	<0.001
TP	0.130	<0.001	0.171	<0.001
ALB	0.210	<0.001	0.097	<0.001
Crea	0.615	<0.001	0.287	<0.001
BUN	0.183	<0.001	0.082	<0.001
WBC	0.227	<0.001	0.105	<0.001
NEUT	0.157	<0.001	0.063	<0.001
EO	0.235	<0.001	0.069	<0.001
RBC	0.463	<0.001	0.133	<0.001
HGB	0.513	<0.001	0.133	<0.001
HCT	0.508	<0.001	0.118	<0.001
MCV	–0.033	0.006	–0.049	<0.001
MCH	0.100	<0.001	–0.006	0.630
MCHC	0.230	<0.001	0.083	<0.001

*^a^P value determined by spearman correlation analysis with respect to the serum UA level.*

*^b^P value determined by partial correlation analysis with respect to the serum UA level adjusted for sex and age.*

### The Association of Hyperuricemia With Overweight/Obesity and Metabolic Syndrome-Related Components

Since our results showed that UA was weakly correlated with many laboratory indexes, we finally clarified the specific association between hyperuricemia and BMI as well as metabolic syndrome-related components (Table 4). The results showed that hyperuricemia was positively associated with increased BMI in all models [Model 3: OR: 1.66 (1.32–2.09), *P* < 0.0001]. In terms of each metabolic disorders, after adjusting for other confounding factors, including all of the metabolic indicators except itself, hyperuricemia had a significant positive association with hypertension [OR: 1.22 (1.03–1.46), *P* = 0.0233] and hypertriglyceridemia [OR: 1.56 (1.21–2.02), *P* = 0.0006], while it had no significant association with hyperglycemia and low HDL-C (all *P* > 0.05).

**TABLE 4:** The association between hyperuricemia and overweight/obese and metabolic dysfunction in all subjects.

Metabolic syndrome-related components		OR (95%CI)	*p* value
BMI ≥ 23 kg/m^2^	Model 1	3.47 (2.83–4.24)	<0.0001
	Model 2	2.81 (2.28–3.46)	<0.0001
	Model 3^a^	1.66 (1.32–2.09)	<0.0001
Elevated BP	Model 1	1.92 (1.65–2.23)	<0.0001
	Model 2	1.77 (1.51–2.08)	<0.0001
	Model 3^b^	1.22 (1.03–1.46)	0.0233
Elevated FPG	Model 1	1.14 (0.92–1.41)	0.2468
	Model 2	1.04 (0.83–1.29)	0.7547
	Model 3^c^	1.00 (0.74–1.34)	0.9799
Elevated TG	Model 1	3.23 (2.77–3.77)	<0.0001
	Model 2	2.69 (2.29–3.15)	<0.0001
	Model 3^d^	1.56 (1.21–2.02)	0.0006
Low HDL-C	Model 1	1.25 (1.07–1.47)	0.0057
	Model 2	1.44 (1.22–1.70)	<0.0001
	Model 3^e^	1.16 (0.92–1.47)	0.2052

*Model 1 was unadjusted; Model 2 was adjusted for Age and Sex; Model 3 was adjusted for Age, Sex, HbA1c, TC, LDL-C, ALT, AST, TP, ALB, Cr, BUN, WBC, NEUT, EO, RBC, HGB, HCT, MCV, MCH, MCHC plus SBP, DBP, FPG, TG and HDL-C (a); plus BMI, FPG, TG and HDL-C (b); plus SBP, DBP, TG, HDL-C (c); plus SBP, DBP, FPG, HDL-C (d); plus SBP, DBP, FPG, TG (e).*

## Discussion

Numerous studies have confirmed that overweight/obese people may be accompanied with various metabolic phenotypes (10, 12, 14, 26). In this retrospective study among general population who underwent routine physical examination, we discovered that hyperuricemia as well as the increase of UA level was positively associated with the presence of MHOO, MANW, and MAOO phenotypes.

Hyperuricemia has been reported to be more manifest in male subjects (27), which is similar to our data. In patients with hyperuricemia, deposition of UA in joints and tissues promotes the occurrence of gout and metabolic disorders (28). Consistent with that conclusion, our data showed that participants with hyperuricemia display higher BMI, SBP, DBP, FPG, TG, TC, LDL-C levels, and lower HDL-C levels when compared with subjects with normouricemia. Our results found that MAOO phenotype was significantly more prone to occur in participants with hyperuricemia in both men and women. These results suggested that hyperuricemia may be closely associated with metabolic disorders, obesity, and its phenotypes, and their interactions may appear to be complex.

Previous studies have shown that obesity and its phenotypes are significantly associated with the risk of hyperuricemia, and the relationship are sex-specific and age-specific differences (29–31). In the Chinese population, Tian et al. suggested that the MHOO phenotype was significantly associated with the risk of hyperuricemia only in women and not in men from the China Health and Nutrition Survey (30). Yu et al. found that MUOO, in comparison with MHOO, was significantly associated with hyperuricemia in Chinese adults (29). High serum UA levels have long been considered a potential master conductor in metabolic syndrome and fat storage (17, 32), but it is still unknown whether serum UA is a risk factor of obesity-related metabolic phenotypes in Chinese population. Thus, our study analyzed the effect of hyperuricemia on various obesity phenotypes from comprehensive perspective.

In an Austrian study, serum UA was a significant predictor of unhealthy obesity in juveniles and adults (22). However, our results have shown that the presence of hyperuricemia or the increase of UA level was associated with the risk of MHOO, MANW, and MAOO phenotypes, and the association did not differ by sex and age, but have some different characteristics among different sex-based and age-based subgroups. As we all know, people with various obesity metabolic phenotypes have different metabolic characteristics, risk, mortality of disease, and quality of life (9, 11, 33). Many studies have shown that hyperuricemia has emerged as a risk marker for clinical diseases including cardiovascular disease, chronic kidney disease, metabolic disease, and premature mortality (34). Thus, we speculated that the association between elevated UA and different obesity phenotypes may be a contributory causal factor for progress of various obesity phenotypes. In addition, features of obesity phenotypes in different sex and age have shown complex results according to previous studies (35, 36). Our study also reflected that the effect of hyperuricemia on different metabolic phenotypes was quite different in both sex and different age subgroups. Taken together, further research on the association between hyperuricemia and obesity phenotypes in different population is significant and indispensable for clinically individualized therapeutic approaches.

The present study further showed that hyperuricemia was significantly associated with obesity phenotypes-related variables such as elevated TG and elevated BP but not elevated FPG and low HDL-C, which is slightly different from other studies. Many epidemiological studies have demonstrated a strong association between UA and various metabolic syndrome-related components, but the association could be differed by study objects (37–39). Nejatinamini et al. found that UA was positively correlated with triglycerides, and negatively with HDL-C in 101 non-smoking Iranians (39). Tian et al. suggested that elevated serum UA concentration was shown to be associated with central obesity and hypertension in middle aged and elderly Chinese (38). In addition, a prospective study demonstrated that serum UA predicts incident metabolic syndrome in the elderly in an analysis of the Brisighella Heart Study (40). Review has concluded that the underlying biological mechanism between hyperuricemia and obesity phenotypes-related components is closely associated with oxidative stress and inflammatory induced by elevated serum UA (32). Compared to previous studies, subjects in the present study were the physical examination population, which suggested that the relationship between hyperuricemia and obesity phenotypes-related variables may have their own characteristics in different populations. Meanwhile, it is time to recommend definitive, large-scale clinical trials to determine whether lowering UA can be beneficial in the treatment and prevention of overweight/obesity, hypertension, dyslipidemia, and insulin resistance.

Some highlights of this study are worth mentioning. Our study is a comprehensive analysis of a large cohort with the major anthropometric data, and conventional biochemical and hematological parameters. The combined use of multiple regression, stratification analysis, and interaction analysis can ensure the reliability of the conclusion. Moreover, it was found that hyperuricemia was positively associated with MHOO, MANW, and MAOO phenotypes to varying degrees and these relationships were not affected by sex or age, which could be expected to provide a theoretical basis for clinical application.

There are some drawbacks of our study that are worth considering. First, as a retrospective cross-sectional study, we were unable to determine the causal relationship between hyperuricemia and obesity phenotype. Second, we did not have access to the body fat content of the participants for some reasons, so a more accurate metabolic classification of obesity is not possible. Third, the data of dietary intake (e.g., intake of foods rich in the sources of UA, intake of saturated and unsaturated fatty acids) were not available due to some reasons. Fourth, the results of sex stratification may be partially affected by menstruation, menopause, and contraceptives (41–43), data of which we did not obtain due to the lack of patient inquiry in health examination. In future researches, we may also collect data on inflammatory cytokines and saturated fat to further explore the potential relationship among UA, inflammation, and the metabolic phenotypes of obesity.

## Conclusion

In our study, hyperuricemia was positively associated with MHOO, MANW, and MAOO phenotypes, and these relationships were not affected by sex or age. Moreover, hyperuricemia was associated differently with various overweight/obesity-related metabolic disorders in the Chinese general population. To sum up, our research provided theoretical basis on the important role of UA for the different metabolic status among Chinese general population, and there needs to be more researches to study the degree of role in different populations for precise prevention and treatment.

## Data Availability Statement

The raw data supporting the conclusions of this article will be made available by the authors, without undue reservation.

## Ethics Statement

The study involving human participants were reviewed and approved by the Ethics Committee of Second Xiangya Hospital of Central South University. Written informed consent for participation was not required for this study in accordance with the national legislation and the institutional requirements.

## Author Contributions

LT and HT designed the study and revised the manuscript. XF, YY, HX, SZ, YF, YD, PJ, HC, and HT conducted the research. XF, YY, and HT analyzed the data. XF, YY, and HX wrote the manuscript. All authors read and approved the final manuscript.

## Conflict of Interest

The authors declare that the research was conducted in the absence of any commercial or financial relationships that could be construed as a potential conflict of interest.

## Publisher’s Note

All claims expressed in this article are solely those of the authors and do not necessarily represent those of their affiliated organizations, or those of the publisher, the editors and the reviewers. Any product that may be evaluated in this article, or claim that may be made by its manufacturer, is not guaranteed or endorsed by the publisher.

## Footnotes

1
www.empowerstats.com

2
http://www.R-project.org

## Supplementary Material

Supplementary Figure 1 Flow chart of the study population.
