# Supplementary figures and images for: The Association Between Hyperuricemia and Obesity Metabolic Phenotypes in Chinese General Population: A Retrospective Analysis
Source: Front Nutr. 2022 Apr 18;9:773220. doi: 10.3389/fnut.2022.773220 (PMC9063096; doi:10.3389/fnut.2022.773220)

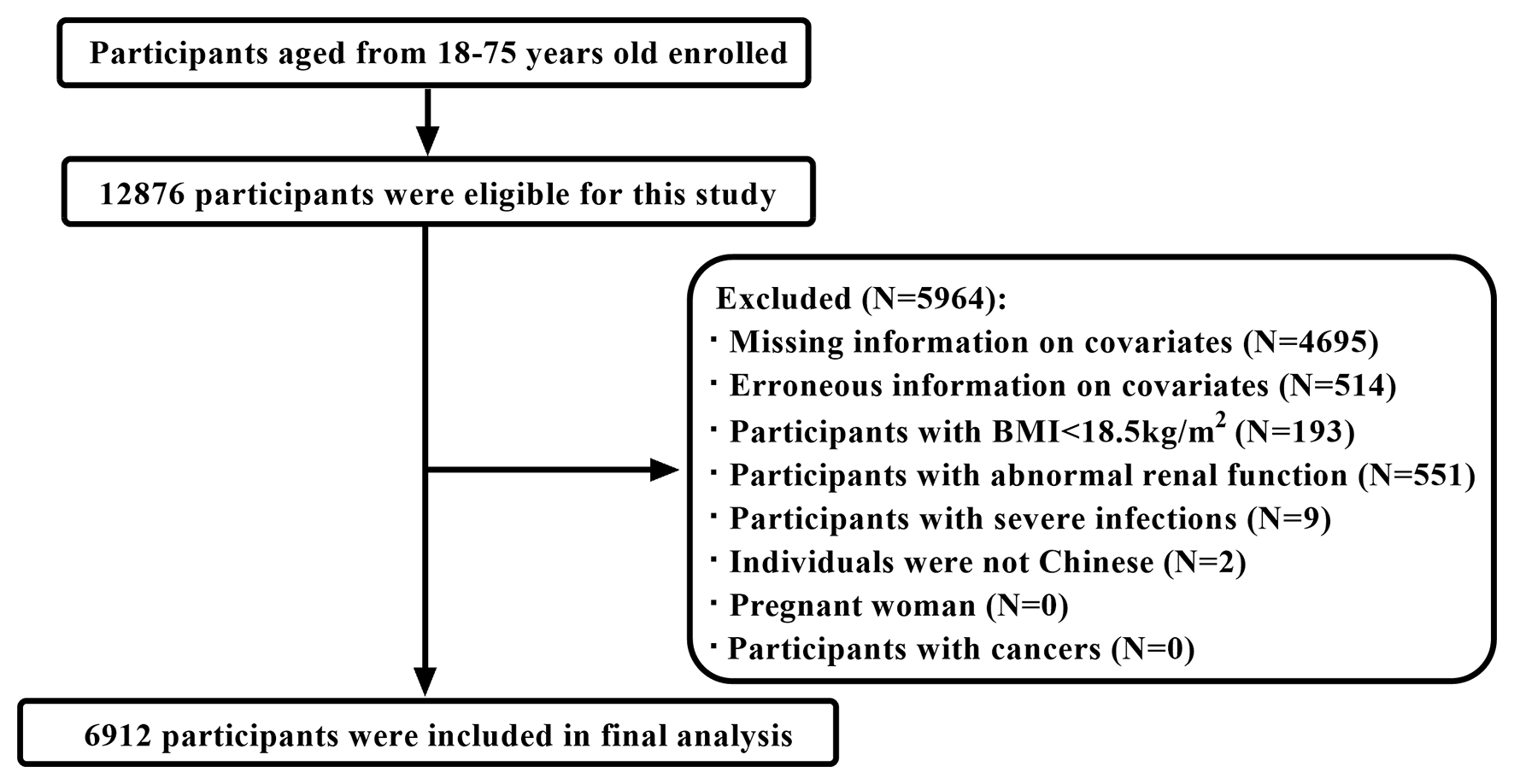

Supplement: Supplementary Figure 1 — Flow chart of the study population. [file Image_1.TIF]
